# Sensitive Aflatoxin M1 Detection in Milk by ELISA: Investigation of Different Assay Configurations

**DOI:** 10.3390/toxins16120515

**Published:** 2024-11-29

**Authors:** Dimitra Kourti, Michailia Angelopoulou, Panagiota Petrou, Sotirios Kakabakos

**Affiliations:** 1 Immunoassays/Immunosensors Lab, Institute of Nuclear & Radiological Sciences & Technology, Energy & Safety, NCSR “Demokritos”, 15341 Aghia Paraskevi, Greece; d.kourti@rrp.demokritos.gr (D.K.); mikangel@ipta.demokritos.gr (M.A.); skakab@rrp.demokritos.gr (S.K.); 2 Analytical Chemistry Lab, Department of Chemistry, University of Athens, Panepistimiopolis Zografou, 15771 Athens, Greece

**Keywords:** aflatoxin M1, ELISA, milk safety, immunoassay

## Abstract

Aflatoxin M1 (AFM1) exposure through dairy products is associated with adverse health effects, including hepatotoxicity and carcinogenicity. Therefore, the AFM1 presence in milk and dairy products is strictly regulated. In this context, the current work focuses on the investigation of different competitive enzyme immunoassay configurations for the determination of AFM1 in milk with high sensitivity and short assay duration. Amongst the configurations tested, the one based on incubation of the anti-AFM1 specific antibody along with the calibrators/samples and a biotinylated conjugate of AFM1 with bovine serum albumin (BSA) in microwells coated with a secondary antibody provided a six-fold lower detection limit than the configuration involving immobilized AFM1-BSA conjugate and liquid phase antibody. The detection limit achieved was 5.0 pg/mL, with a dynamic range of up to 2.0 ng/mL. The assay was repeatable with intra- and inter-assay coefficients of variations lower than 3.2% and 6.5%, respectively, and accurate with recovery values from 87.5 to 108%. Moreover, the assay was completed in 1.5 h. The excellent analytical characteristics and short analysis time make the proposed assay suitable for use by the food industry. Furthermore, the proposed configuration could be employed to enhance the detection sensitivity of competitive immunoassays for other low-molecular-weight analytes.

## 1. Introduction

Mycotoxins, which are low-molecular-weight compounds produced by fungi, are categorized among the most hazardous naturally occurring chemical substances in food and feed. So far, over 300 mycotoxins have been identified, most of them produced by species of *Aspergillus*, *Fusarium*, and *Penicillium* fungi [1,2,3]. Several factors contribute to crop contamination with mycotoxins, including improper agricultural practices during harvest, storage, or transport, as well as high humidity before and after harvest, which promotes fungal growth [4]. Mycotoxins can enter the human body through the direct consumption of plant-based foods contaminated during cultivation and/or storage or via the ingestion of mycotoxin metabolites present in animal-derived products such as milk or eggs. When ruminants (cows, goats, sheep, etc.) ingest feed contaminated with aflatoxin B1 (AFB1), their livers metabolize it into aflatoxin M1 (AFM1) by the action of hepatic microsomal cytochrome P450 [5]. AFM1, which is then excreted in their milk, poses a significant health risk to humans, as AFM1 has been shown to suppress the immune system, induce alterations in DNA structure, and, at high levels, lead to severe conditions like liver cirrhosis, hepatocellular carcinoma, and stunted growth in infants [6].

Eliminating AFM1 from dairy products is challenging because it remains stable after thermal treatments like pasteurization or sterilization, which typically occur at much lower temperatures than those needed to break down AFM1 (237–306 °C) [7,8]. Proposed methods to remove AFM1 from milk include treatment with chemical agents like acids or enzymes, which are impractical for application by the food industry [9]. Thus, the main way to protect public health is to monitor AFM1 levels throughout the production of dairy products. The European Union has set the maximum allowable limit for AFM1 in milk for infant consumption at 25 pg/mL and for adult consumption at 50 pg/mL, which are amongst the lower limits worldwide [10].

Various methods have been developed to detect AFM1 in milk and dairy products. Chromatographic techniques like high-performance liquid chromatography (HPLC) with various detectors (e.g., mass spectrometry [11], absorbance [12], or fluorescence detectors [13,14,15]) offer high specificity and sensitivity but are expensive and require complex sample preparation. Immunochemical methods, such as enzyme-linked immunosorbent assays (ELISA) [16,17,18], immunochromatographic strips [19,20], and immunosensors [21,22], are also used for AFM1 detection. Immunochromatographic strips are fast and suitable for field analysis but provide qualitative or semi-quantitative results. Immunosensors, while highly sensitive and fast, are still in the development phase and are more suitable for point-of-need applications. Thus, ELISA remains the preferred method for laboratory analysis due to its ease of use, high sensitivity, and ability to process multiple samples in a short time. There are a few commercially available ELISA kits for AFM1 determination in foods [16,17,18] with detection limits and working ranges that cover the maximum allowable limits set by the regulatory authorities, although some of them require time- and labor-consuming sample treatments in order to achieve the required analytical performance and reduce matrix effects. In addition, there are reports [23,24,25,26,27] of novel approaches for AFM1 determination in unprocessed or minimally processed milk samples exploiting new antibodies and/or detection methods to improve detection sensitivity and minimize assay duration.

**Figure 1:**
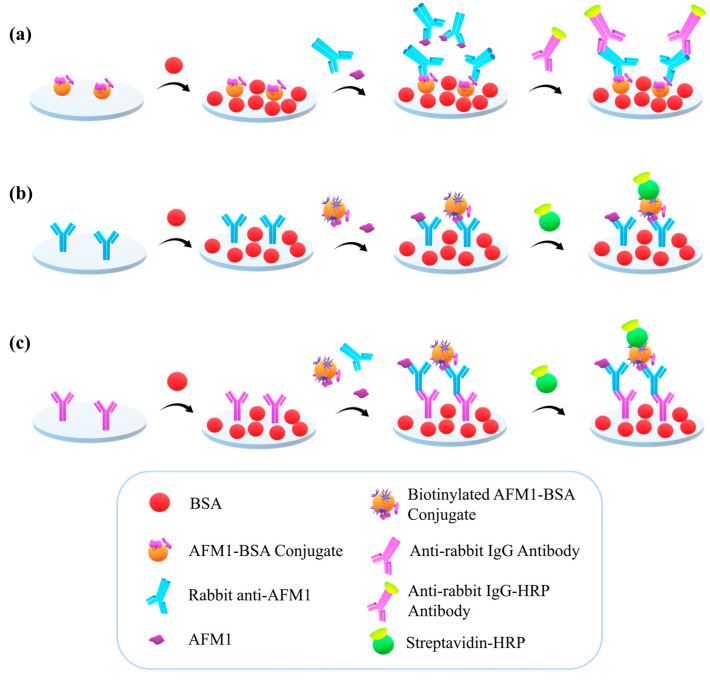
Schematic of the different competitive enzyme immunoassay configurations for AFM1 detection in milk involving (**a**) the immobilization of AFM1-BSA conjugate followed by reaction with rabbit polyclonal anti-AFM1 antibody and detection with goat anti-rabbit IgG antibody-HPR conjugate (anchored antigen configuration), (**b**) the immobilization of rabbit polyclonal anti-AFM1 antibody and detection with biotinylated AFM1-BSA conjugate and streptavidin-HRP (anchored primary antibody configuration), and (**c**) the immobilization of goat anti-rabbit IgG antibody followed by reaction with a mixture of rabbit polyclonal anti-AFM1 antibody and biotinylated AFM1-BSA conjugate and detection with streptavidin-HRP (anchored secondary antibody configuration).

In this work, three different competitive enzyme immunoassay configurations (Figure 1) were evaluated for their ability to detect AFM1 in milk, aiming to define the one providing the highest sensitivity. All configurations used a rabbit polyclonal antibody against AFM1 and an AFM1 conjugate with bovine serum albumin (AFM1-BSA). In the first configuration, which is referred to as an anchored antigen configuration, the AFM1-BSA conjugate was immobilized to the microtiter wells and competed with the AFM1 in the calibrators for binding to the antibody (Figure 1a). A secondary antibody (goat anti-rabbit IgG antibody) labeled with horseradish peroxidase (HRP) was used to detect the immunocomplexes formed on the wells. In the second configuration, termed as anchored primary antibody configuration, the rabbit polyclonal anti-AFM1 was adsorbed to the microtiter wells and the competition was performed between the AFM1 in the calibrators and a biotinylated AFM1-BSA conjugate (Figure 1b). In this configuration, a streptavidin-HRP conjugate was used for detection. Lastly, in the third configuration, a secondary antibody was immobilized to the microtiter wells, to bind the rabbit polyclonal anti-AFM1 antibody from a mixture with the AFM1 calibrator and the biotinylated AFM1-BSA conjugate (Figure 1c). This configuration will be referred to as an anchored secondary antibody configuration. The detection of the immunocomplexes formed was performed using the streptavidin-HRP conjugate. After the optimization of several conditions for each one of the different configurations, they were compared in terms of repeatability and sensitivity of the measurements. The configuration that provided the highest detection limit and lowest intra- and inter-assay variation was further evaluated in terms of accuracy and compared with enzyme immunoassays reported in the literature for the detection of AFM1 in milk and other dairy products.

## 2. Results and Discussion

### 2.1. Optimization of the Assays

Each one of the three competitive enzyme immunoassay configurations presented in Figure 1 has been optimized so as to achieve the highest possible detection sensitivity.

#### 2.1.1. Anchored Antigen Configuration

**Figure 2:**
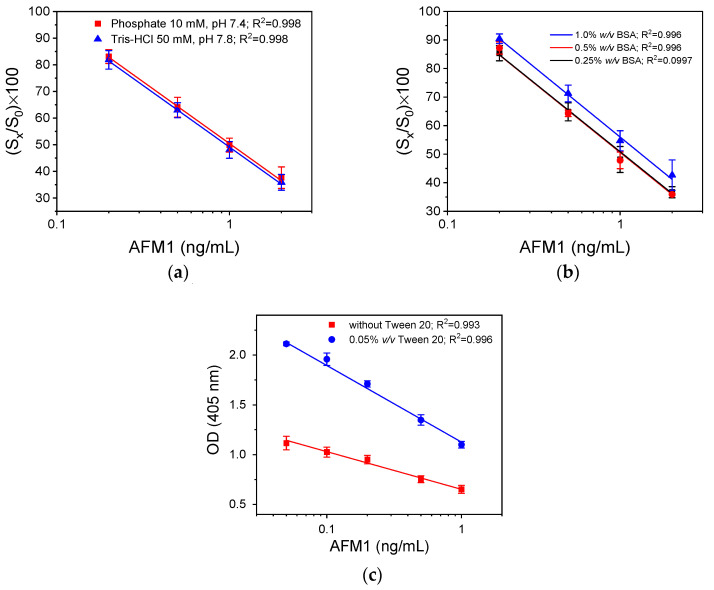
(**a**) AFM1 calibration curves received using as primary immunoreaction buffer 10 mM PBS, pH 7.4, 0.5% (*w*/*v*) BSA (red line), or 50 mM Tris-HCl, pH 7.8, 0.9% (*w*/*v*) NaCl, 0.5% (*w*/*v*) BSA (blue line). (**b**) AFM1 calibration curves received using as primary immunoreaction buffer 50 mM Tris-HCl, pH 7.8, 0.9% (*w*/*v*) NaCl, containing 0.25% (black line), 0.5% (red line), or 1.0% (*w*/*v*) BSA (blue line). (**c**) AFM1 calibration curves received using as primary immunoreaction buffer 50 mM Tris-HCl, pH 7.8, 0.9% (*w*/*v*) NaCl, 0.5% (*w*/*v*) BSA with 0.05% (*v*/*v*) Tween 20 (blue line) or without Tween 20 (red line). In all cases, the duration of the primary immunoreaction was 1 h, and the duration of the reaction with the HRP-labeled secondary antibody was 40 min. Each point corresponds to the mean value of 3 replicate measurements ± SD.

The optimization of the anchored antigen configuration started with the selection of primary immunoreaction buffer, i.e., the buffer used for the preparation of rabbit anti-AFM1 solution. More specifically, two different buffers were tested, 50 mM Tris-HCl, pH 7.8, 0.9% (*w*/*v*) NaCl, 0.5% (*w*/*v*) BSA, and 10 mM phosphate buffer saline (PBS), pH 7.4, 0.9% (*w*/*v*) NaCl, 0.5% (*w*/*v*) BSA. As shown in Figure 2a, the calibration curves obtained with the two solutions were identical; however, the analytical signal received was approximately 15% higher when the Tris-HCl buffer was used compared with that obtained with the PBS buffer; therefore the Tris-HCl buffer was selected for further experimentation. The optimal BSA concentration in the primary immunoreaction buffer was also determined through calibration curves obtained with 50 mM Tris-HCl buffer, pH 7.8, containing 0.25, 0.5, or 1.0% (*w*/*v*) BSA. From the curves shown in Figure 2b, it can be concluded that the highest sensitivity was achieved for the BSA concentration in the assay buffer equal to or lower than 0.5% (*w*/*v*) BSA. Furthermore, the effect of the addition of Tween 20 to the primary immunoreaction buffer at a concentration of 0.05% (*v*/*v*) on the signal and the calibration curve was investigated. It was found (Figure 2c) that the analytical signal almost doubled when the buffer containing Tween 20 was used compared to that received with the buffer not containing Tween 20, while the sensitivity of the assay was not affected. Based on these results, 50 mM Tris-HCl, pH 7.8, 0.9% (*w*/*v*) NaCl, 0.5% (*w*/*v*) BSA, 0.05% (*v*/*v*) Tween 20, was selected as the primary immunoreaction buffer for the three immunoassay configurations investigated.

**Figure 3:**
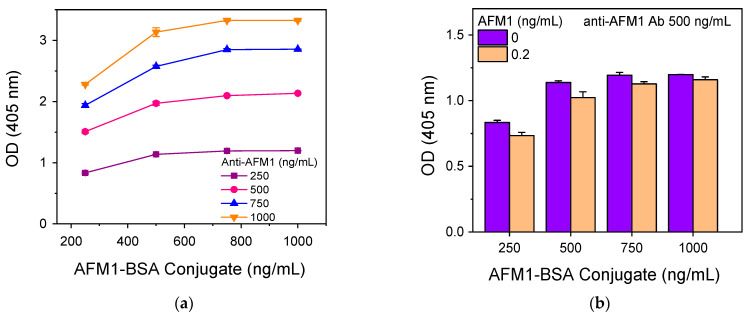
(**a**) Net optical absorbance values at 405 nm obtained for different concentrations of AFM1-BSA conjugate and anti-AFM1 antibody. (**b**) Net optical absorbance values at 405 nm obtained for the zero calibrator (purple columns) and a calibrator containing 0.2 ng/mL AFM1 (orange columns) versus the AFM1-BSA concentration used for coating. The antibody concentration was 500 ng/mL; the primary immunoreaction lasted 1 h and the reaction with the HRP-labeled secondary antibody 40 min. Each point corresponds to the mean value of 3 replicate measurements ± SD.

Following the selection of the optimal primary immunoreaction solution, the concentrations of the AFM1-BSA conjugate for immobilization to the microtiter wells and the anti-AFM1 antibody were investigated to determine the combination leading to adequate analytical signal and high detection sensitivity. As shown in Figure 3a, for the AFM1-BSA conjugate concentrations used for coating, maximum plateau signal values were obtained at concentrations equal to or higher than 750 ng/mL. Nonetheless, a sufficient net signal is obtained using an anti-AFM1 antibody concentration equal to 500 ng/mL for all AFM1-BSA conjugate concentrations used for coating. Thus, using the antibody at a concentration of 500 ng/mL, the effect of the AFM1-BSA conjugate concentration used for coating on the signal received for calibrators containing 0 and 0.2 ng/mL AFM1 was determined. The results shown in Figure 3b indicate that the highest sensitivity is obtained for the combination of 250 ng/mL AFM1-BSA conjugate and 500 ng/mL anti-AFM1 antibody.

**Figure 4:**
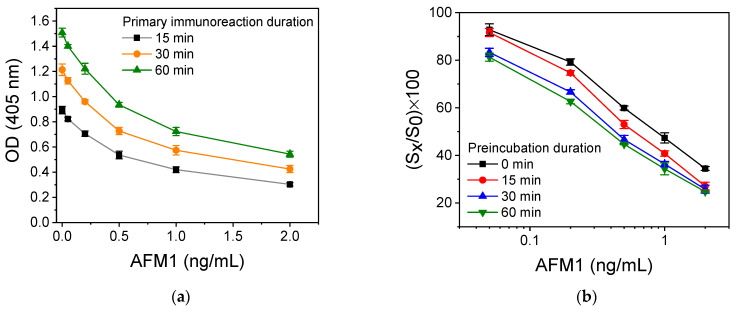
(**a**) Calibration curves obtained for primary immunoreaction duration of 15 (black line), 30 (orange line), or 60 min (green line). (**b**) Calibration curves obtained without (black line) or after 15 (red line), 30 (blue line), or 60 min (green line) preincubation of the anti-AFM1 antibody with the calibrators. The primary immunoreaction duration was 30 min in all cases. Each point corresponds to the mean value of 3 replicate measurements ± SD.

Another parameter optimized was the primary immunoreaction duration. In Figure 4a, the calibration curves obtained for primary immunoreaction durations of 15, 30, and 60 min are provided. As shown, 30 min provided an adequate signal, whereas the sensitivity was the same for the three primary immunoreaction durations tested. The detection limit of the assay was calculated as the concentration corresponding to the mean value of the zero calibrator signal –3SD (*n* = 8). The detection limit achieved for the three primary immunoreaction durations tested was 50 pg/mL, which marginally meets the limit set by the European Union for milk consumed by adults. For this reason, it was investigated whether the sensitivity of the immunoassay might be improved by preincubating the calibrators with the anti-AFM1 antibody prior to incubation with the AFM1-BSA conjugate that has been immobilized onto the wells. In Figure 4b, the calibration curves obtained for preincubation times of 15, 30, and 60 min are depicted along with the curve obtained without preincubation. As shown, the sensitivity of the immunoassay improves as the preincubation time increases by up to 30 min, while prolonging preincubation to 60 min did not further improve the assay sensitivity. Thus, if the calibrators are preincubated with the anti-AFM1 antibody solution for 30 min prior to addition into the AFM1-BSA-coated wells, a detection limit of 30 pg/mL is achieved, with a working range from 50 to 2000 pg/mL. Although this detection limit meets the requirements for AFM1 detection in milk for consumption for adults, it does not satisfy the requirements for infant milk. Therefore, other assay configurations were investigated.

#### 2.1.2. Anchored Primary Antibody Configuration

**Figure 5:**
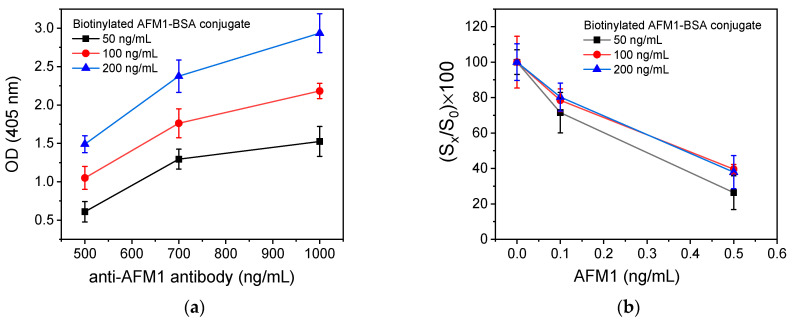
(**a**) Net optical absorbance values at 405 nm obtained for different concentrations of biotinylated AFM1-BSA conjugate and anti-AFM1 antibody. (**b**) Net optical absorbance values at 405 nm corresponding to the zero calibrator and calibrators containing 0.1 and 0.5 ng/mL AFM1 versus the biotinylated AFM1-BSA concentration. The anti-AFM1 antibody concentration in the coating solution was 700 ng/mL. Each point corresponds to the mean value of 3 replicate measurements ± SD.

The anchored primary antibody configuration involved direct immobilization to the microtiter wells of the anti-AFM1 antibody followed by incubation with a mixture of AFM1 calibrators and biotinylated AFM1-BSA conjugate (Figure 1b). Then, a horseradish peroxidase-labeled streptavidin was used for the detection of the immunocomplexes formed in the wells. The optimization of this configuration included the selection of the anti-AFM1 antibody and biotinylated AFM1-BSA concentrations with respect to the zero calibrator signal and detection sensitivity. It was found that an adequate analytical signal (Figure 5a) was provided using a concentration of 700 ng/mL for the antibody and 50 ng/mL for the biotinylated AFM1-BSA conjugate. This combination also provided higher percent inhibition values in the presence of AFM1 (Figure 5b). However, in all cases, the intra-assay coefficient of variation was higher than 7.0%, leading to a limit of detection of approximately 70 pg/mL (Figure S1). This result could be ascribed to the negative effect on antibody functionality upon adsorption onto the well surface. To alleviate this effect, a third configuration was investigated in which the anti-AFM1 antibody was immunoadsorbed on a secondary antibody directly immobilized to the microtiter wells.

#### 2.1.3. Anchored Secondary Antibody Configuration

**Figure 6:**
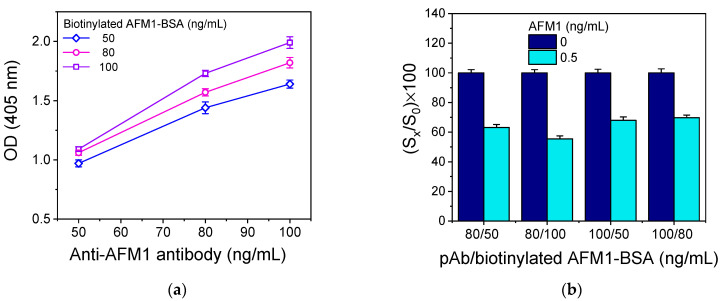
(**a**) Optical absorbance values at 405 nm obtained for different concentrations of biotinylated AFM1-BSA conjugate and anti-AFM1 antibody. (**b**) Percent signal values obtained for a calibrator containing 0.5 ng/mL AFM1 (light blue columns) with respect to the zero calibrator (navy blue columns) for different combinations of the biotinylated conjugate and the anti-AFM1 antibody concentrations. In all cases, the duration of the primary immunoreaction was 60 min, and the reaction with streptavidin-HRP was 30 min. Each point corresponds to the mean value of 3 replicate measurements ± SD.

As with the other two configurations, various immunoassay parameters were optimized, including the concentrations of the anti-AFM1 antibody and the biotinylated AFM1-BSA conjugate and the duration of the different immunoassay steps. In Figure 6a, the net signals obtained for the zero calibrator using different combinations of anti-AFM1 antibody and biotinylated AFM1-BSA conjugate concentrations are presented. The results show that, to achieve an adequate zero calibrator signal, anti-AFM1 antibody concentrations equal to or higher than 80 ng/mL in combination with biotinylated AFM1-BSA conjugate concentrations equal to or higher than 50 ng/mL were required. To determine which combination provides the highest sensitivity, four different combinations providing similar zero calibrator signals were tested. It was found that the highest sensitivity was achieved using the anti-AFM1 antibody at 80 ng/mL and the biotinylated AFM1-BSA conjugate at 100 ng/mL (Figure 6b).

**Figure 7:**
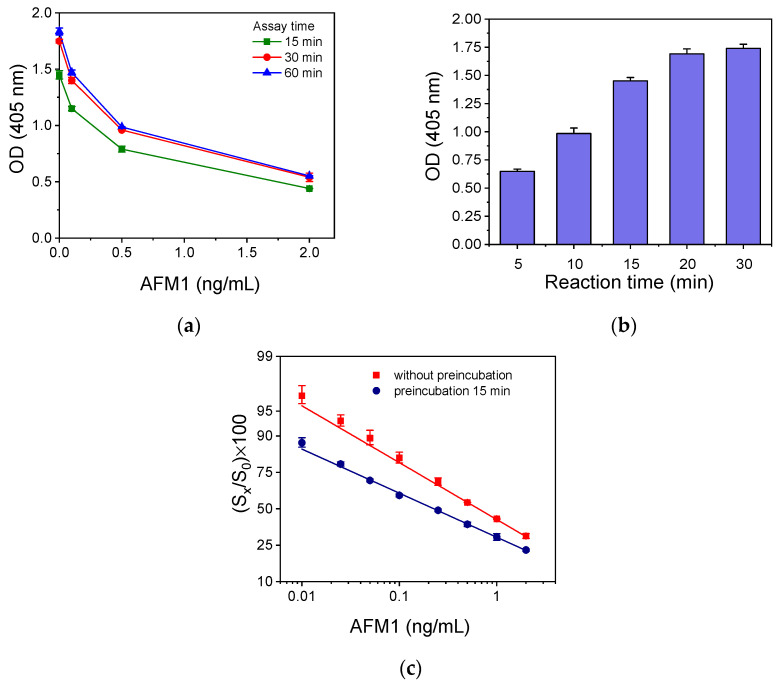
(**a**) Calibration curves obtained for primary immunoreaction duration of 15 (green line), 30 (red line), or 60 min (blue line). (**b**) Optical absorbance values at 405 nm with respect to the duration of reaction with streptavidin-HRP. The immunoassay duration was 30 min. (**c**) Calibration curves obtained without (red line) and with 15 min preincubation (blue line) of AFM1 calibrators with the anti-AFM1 antibody. Each point corresponds to the mean value of 3 replicate measurements ± SD.

The next step was the selection of immunoreaction duration. As shown in Figure 7a, maximum plateau signal values were obtained for immunoreaction duration equal to or higher than 30 min. Additionally, the assay sensitivity was the same for all primary immunoreaction durations tested. The optimum duration of the reaction with the streptavidin-HPR conjugate was also determined. Maximum plateau values were reached after 20 min of reaction, while almost 85% of the maximum signal was reached after 15 min of reaction (Figure 7b). Thus, for the final protocol, a duration of 30 min was selected for the primary immunoreaction and 15 min for the reaction with the streptavidin-HRP conjugate. The detection limit of the assay performed with this protocol was 50 pg/mL. To increase the analytical sensitivity of the assay, a preincubation step of AFM1 calibrators with the anti-AFM1 antibody was added. It was found that a 15 min preincubation led to considerable sensitivity enhancement compared to the assay without preincubation (Figure 7c), whereas longer preincubation times only marginally improved the assay sensitivity. The fact that the preincubation of anti-AFM1 with the AFM1 in the calibrators improved the sensitivity of both the anchored antigen and the anchored secondary antibody configuration is an indication of higher antibody binding affinity toward the AFM1 that is conjugated to protein rather than the free AFM1. This higher binding affinity for the protein-conjugated analyte can be ascribed to the multivalency of the conjugate that facilitates the binding of both antibody sites to the conjugate. Moreover, the reduced preincubation time required when the anchored secondary antibody configuration was followed could be attributed to the increased analyte-to-antibody volume ratio (2:1 *v*/*v*) in the preincubation mixture compared to the 1:1 *v*/*v* ratio employed in the anchored antigen configuration.

### 2.2. Analytical Evaluation of the Selected Immunoassay Configuration

The analytical characteristics of the anchored secondary antibody immunoassay configuration were determined based on the calibration curve presented in Figure 7c (blue circles). More specifically, the detection limit (LOD) achieved was 5.0 pg/mL, which is six times lower than that obtained with the optimized protocol of the anchored antigen configuration. This improvement is due to the fact that, in the anchored secondary antibody configuration, it was possible to achieve an adequate signal using an approximately 12.5 times lower anti-AFM1 antibody concentration and 10 times lower AFM1-BSA concentration per well compared to those implemented in the anchored antigen configuration. Additionally, the use of a lower antibody concentration in the anchored secondary antibody configuration compared to that used in the first one led to a reduction in the required preincubation time from 30 to 15 min. Moreover, the detection limits of the anchored secondary antibody configuration are 5 and 10 times lower than the maximum allowable concentrations of AFM1 set by the EU for infant and adult milk, respectively.

The linear dynamic range of the assay extended from the limit of quantification (LOQ), which was 10 pg/mL to 2000 pg/mL. The intra-assay repeatability was evaluated by determining the coefficient of variation (CV) of the values received for three milk samples spiked with AFM1 to final concentrations of 40, 120, 400, and 1200 pg/mL, analyzed in triplicate on the same day. Similarly, the inter-assay repeatability was calculated by determining the CVs of the values obtained for the same samples analyzed in duplicate on four different days over a period of one month. The intra-assay and the inter-assay CVs were less than 3.2 and 6.5%, respectively, demonstrating the high repeatability of the method. The excellent repeatability achieved with the anchored secondary antibody assay configuration compared to that of the anchored primary antibody configuration is attributed to the fact that the primary antibody is immunoadsorbed to the secondary antibody, and therefore, its functionality is practically unaffected.

The accuracy of the assay was determined through experiments in which known amounts of AFM1 were spiked in three full-fat cow milks produced by different Greek dairy companies (recovery experiments). As is depicted in Table S1, the percent recovery values ranged from 87.5 to 108%, demonstrating the high accuracy of the assay.

### 2.3. Comparison with Other ELISA Methods

A comparison between the developed method and other ELISA methods in terms of the detection limit, working range, and assay duration is presented in Table 1. In all cases, the assay duration mentioned includes the preincubation time of the samples, the immunoreaction, and the duration of the subsequent signal generation/amplification steps (i.e., the incubation of the wells with the chromogenic enzyme substrate). Radoi and his colleagues investigated different competitive immunoassay configurations to detect AFM1 in milk matrices achieving limits of detection ranging from 4 to 12.5 pg/mL [23]. It was shown that the faster and more sensitive immunoassay was obtained by employing superparamagnetic nanoparticles conjugated with protein G as solid support [23]. A sandwich-type electrochemical immunoassay has also been reported that combined an anti-AFM1 antibody-modified electrode and an aptamer against AFM1 conjugated to gold nanoparticles along with a DNA primer sequence as a detection element [24]. The primer initiated a rolling circle amplification (RCA) reaction that produced a long single-stranded DNA, which folded into a peroxidase-mimicking DNAzyme in the presence of hemin and potassium cations. The DNAzyme oxidized 2-aminophenol to 3-aminophenylhydrazine, in presence of H_2_O_2_, and the latter was reduced onto the electrode providing the analytical signal. Despite the elaborative signal amplification scheme that resulted in a long assay duration (~6.5 h), the detection limit achieved was 150 pg/mL [24]. More recently, Cai et al. developed a competitive enzyme immunoassay using an anti-idiotypic nanobody named VHH C4 that could bind AFM1, providing a detection limit of 50 pg/mL when employed as a solid-phase reagent [25]. When VHH C4 was used as a surrogate to replace AFM1 in the calibrators and to compete with the AFM1-BSA immobilized antigen for binding to a monoclonal antibody, the assay sensitivity was improved reaching a detection limit of 35 pg/mL [26]. Finally, a monoclonal antibody developed in plant cells was evaluated as a solid-phase reagent in a competitive immunoassay for the determination of AFM1, providing a detection limit of 3.0 pg/mL [27]. It should be noted that all the above-discussed methods have been employed for AFM1 detection in milk or milk products. The milk was used either without any pretreatment [24] or after centrifugation to remove fat prior to testing [23,25,26,27].

Compared to the literature methods, the assay proposed here is listed amongst the most sensitive and fast ones. The employment of biotinylated AFM1-BSA conjugate as a competitor of the free analyte played a significant role in the reduction in both the antibody and the conjugate concentrations. This reduction was feasible due to the presence of multiple biotin moieties in the AFM1-BSA conjugate, which led to binding of more than one streptavidin-HRP molecule per molecule of analyte, increasing the absolute signal. Moreover, the fact that the reaction takes place primally in the liquid phase helps to further reduce the concentrations of antibody and AFM1-BSA conjugate due to the enhanced diffusion of immunoreagents. The decreased antibody and biotinylated AFM1-BSA conjugate concentrations favored the coupling of the free analyte to the binding sites of the antibody over the AFM1-BSA conjugate, thus increasing the assay sensitivity.

A significant advantage of the method developed over the literature methods is the wide linear dynamic range, which means that samples with an AFM1 concentration up to 2.0 ng/mL (as those reported in reference [15,16]) can be analyzed directly without sample dilution.

## 3. Conclusions

The contamination of milk with AFM1 is a global problem, as the crops used for animal feeding are susceptible to fungi development both before and after harvest. One could claim that the most efficient way to avoid milk contamination with AFM1 would be to monitor the presence of mycotoxins in the feed. However, although feeds are regularly tested, AFM1 is still detected in milk according to the relevant literature reports [6,7]. Thus, dairy companies are obliged to determine AFM1 in milk upon arrival at processing facilities as part of their routine quality control. Due to the tendency of milk for spoilage, the quality control should be completed as fast as possible. To address this need, in the present study, we explored three different competitive immunoassay configurations to achieve highly sensitive detection of AFM1 in milk samples. Among these, the configuration that provided the lowest detection limit involved the immobilization of a secondary antibody onto the microtiter wells. This configuration allowed the AFM1 in the sample to compete with a biotinylated AFM1-BSA conjugate for the binding sites of the anti-AFM1 antibody. Sensitivity was significantly improved by preincubating the anti-AFM1 antibody with samples before introducing the mixture to the wells and adding the biotinylated AFM1-BSA conjugate. This approach achieved the impressive detection limit of 5 pg/mL, which is 10 and 5 times lower than the maximum allowable limits set by the EU for AFM1 in milk for adults and infants, respectively. It should be noted that the EU legislation limits for AFM1 are amongst the lower allowable limits worldwide. The working range of the assay (0.01–2.0 ng/mL) outperformed several literature methods and commercial ELISA kits, and the analysis was completed within 90 min, including a brief 15 min preincubation step. The assay demonstrated high repeatability, with intra-assay and inter-assay coefficients of variation below 3.2% and 6.5%, respectively, and accuracy, with recovery rates between 87.5% and 108%. Moreover, this assay required no pretreatment of milk samples. With its outstanding analytical performance, short duration, and minimal sample pretreatment, this assay shows strong promise for routine AFM1 screening in milk. Furthermore, the immunoassay configuration proposed here could be adapted to develop highly sensitive competitive immunoassays for other toxins and low-molecular-weight analytes in general, broadening its potential applications.

## 4. Materials and Methods

### 4.1. Materials

AFM1, bovine serum albumin (BSA), AFM1-BSA conjugate, polyclonal anti-rabbit IgG antibody developed in goat conjugated with HRP, polyclonal anti-rabbit IgG antibody developed in goat, streptavidin conjugated to horseradish peroxidase (streptavidin-HRP), 2′,2-azino-bis (3-ethylbenzothiazoline-6-sulphonic acid) (ABTS), and Tween 20 were obtained from Merck KGaA (Darmstadt, Germany). Ninety-six-well microtiter plates were from Greiner Diagnostic GmbH (Bahlingen, Germany). The biotinylation of AFM1-BSA conjugate was performed using 3-sulfo-succinimidyl-6-[biotinamido] (Thermo Fisher Scientific; Waltham, MA, USA) according to a previously published protocol [28]. The rabbit polyclonal anti-AFM1 antibody was purchased from AntiProt (Puchheim, Germany). All other chemicals and reagents were from Merck KGaA (Darmstadt, Germany). The water used in all experiments was doubly distilled. Pasteurized full-fat cow milk (3.5% fat) from three different Greek dairy companies (Larisa Dairy S.A. “OLYMPUS”, Larisa, Greece; DELTA FOODS S.A. Agios Stefanos, Attica, Greece; EVOL—AGROTIKOS SYNETERISMOS VOLOU, Volos, Greece) was purchased at the local market. All purchased milk samples were tested with the AgraQuant^®^ Aflatoxin M1 High Sensitivity ELISA kit (Romer Labs GmbH; Butzbach, Germany) to confirm the absence of detectable amounts of AFM1. The optical density of the wells at 405 nm was measured using the Victor 3 1420 Multilabel Counter (PerkinElmer; Waltham, MA, USA).

### 4.2. Buffers

The buffers used were the following: (a) coating buffer: 50 mM carbonate buffer, pH 9.2; blocking buffer: 10 mg/mL BSA solution in 0.1 M NaHCO_3_, pH 8.5; washing buffer A: 10 mM Tris-HCl, pH 8.25, 0.9% (*w*/*v*) NaCl; washing buffer B: washing buffer A with 0.5 mL/L Tween 20; assay buffer: 50 mM Tris-HCl, pH 7.8, 0.9% (*w*/*v*) NaCl, 0.5% (*w*/*v*) BSA, 0.05% (*v*/*v*) Tween 20; secondary antibody buffer: 0.1 M Tris-HCl buffer, pH 8.25, 0.5% (*w*/*v*) BSA, 0.9% (*w*/*v*) NaCl; streptavidin buffer: 50 mM phosphate buffer, pH 6.5, 0.9% (*w*/*v*) NaCl, 1% (*w*/*v*) BSA; HRP substrate solution: 0.03% *v*/*v* H_2_O_2_ and 1.9 µM ABTS in 0.1 M citrate-phosphate buffer, pH 4.5.

### 4.3. AFM1 Enzyme Immunoassay Configurations

#### 4.3.1. Anchored Antigen Configuration Protocol

Microtiter wells were coated overnight at room temperature (RT) with 100 μL of a 250 ng/mL AFM1-BSA conjugate solution in coating buffer. Then, the wells were washed twice with 300 μL per well of washing buffer A, and 300 μL of blocking buffer were added for 2 h at RT. AFM1 calibrators prepared in milk were preincubated for 30 min at a 1:1 volume ratio with a 500 ng/mL rabbit anti-AFM1 antibody solution in assay buffer. After washing the wells as previously, 100 μL of the preincubated mixtures were added per well and incubated for 30 min under shaking. Then, the wells were washed 4 times with 300 μL of washing buffer B, and 100 μL of a 1.0 μg/mL anti-rabbit IgG-HRP conjugate solution in secondary antibody buffer were added per well and incubated under shaking for 40 min. Finally, after washing 4 times with 300 μL of washing buffer B, the wells were incubated with 100 μL of HRP substrate solution for 30 min under shaking at RT in the dark. The mean optical density value of the AFM1 calibrators (S_x_) was expressed as a percentage of the zero calibrator optical density value (S_0_) and plotted versus the AFM1 concentration in the calibrators to construct the calibration curve.

#### 4.3.2. Anchored Primary Antibody Configuration Protocol

Microtiter wells were coated through overnight incubation at RT with 100 μL per well of a 700 ng/mL rabbit polyclonal anti-AFM1 antibody solution in coating buffer. After washing 2 times with 300 μL per well with washing buffer A, the wells were blocked through incubation for 2 h at RT with 300 μL of blocking buffer. Then, the wells were washed as previously, and 50 μL of AFM1 calibrators in milk and 50 μL of a 50 ng/mL biotinylated AFM1-BSA conjugate solution in assay buffer were added per well and incubated for 30 min under shaking. After that, the wells were washed 4 times with 300 μL of washing buffer B, and 100 μL of a 250 ng/mL streptavidin-HRP solution in streptavidin buffer were added per well and incubated under shaking for 30 min. Finally, after washing 4 times with 300 μL of washing buffer B, the wells were incubated with 100 μL of HRP substrate solution for 30 min under shaking at RT in the dark. The construction of the calibration curve was performed as described in Section 4.3.1.

#### 4.3.3. Anchored Secondary Antibody Configuration Protocol

Microtiter wells were coated through overnight incubation at RT with 100 μL per well of a 5 μg/mL goat anti-rabbit IgG antibody solution in coating buffer. After washing 2 times with 300 μL per well of washing buffer A, the wells were blocked through incubation for 2 h at RT with 300 μL of blocking buffer and washed as previously. For the assay, AFM1 calibrators in milk were preincubated for 15 min at a 2:1 volume ratio with an 80 ng/mL rabbit anti-AFM1 antibody solution in assay buffer. Then, 75 μL of the preincubated anti-AFM1 antibody/AFM1 calibrator mixtures and 25 μL of a 100 ng/mL biotinylated AFM1-BSA conjugate solution in assay buffer were added per well. After 30 min of incubation at RT under shaking, the wells were washed 4 times with 300 μL of washing buffer B, and 100 μL of a 250 ng/mL streptavidin-HRP solution in streptavidin buffer were added per well and incubated under shaking for 15 min. Finally, after washing 4 times with 300 μL of washing buffer B, the wells were incubated with 100 μL of HRP substrate solution for 30 min under shaking at RT in the dark. The construction of the calibration curve was performed as described in Section 4.3.1.

## Figures and Tables

**Table 1:** Comparison of the assay developed with other ELISA methods for determination of AFM1 in milk and dairy products.

Assay Configuration	Sample	LOD (pg/mL)	Dynamic Range (pg/mL)	Assay Duration	Ref.
Indirect competitive ELISA	defatted milk milk powder	8.0	8.0–500	120 min	[23]
Direct competitive ELISA		12.5	12.5–100	90 min	
Direct competitive ELISA based on superparamagnetic nanoparticles modified with protein G		4.0	4.0–250	55 min	
Direct competitive ELISA based on superparamagnetic nanoparticles modified with anti-mouse IgG antibody		8.0	8.0–125	55 min	
Sandwich electrochemical ELISA using aptamer-AuNPs-DNA primer	skim milk milk powder milk beverage	150	500–80,000	~6.5 h	[24]
Competitive ELISA using a nanobody	defatted milk yogurt milk powder	50	100–600	145 min	[25]
Indirect Competitive ELISA using a nanobody as binder and as surrogate standard	defatted milk yogurt milk powder	35	45–329	145 min	[26]
Competitive ELISA using a plant-derived antibody	milk	3.0	5.0–110	75 min	[27]
Competitive ELISA with immobilized secondary antibody and primary immunoreaction in liquid phase	milk	5.0	10–2000	90 min	This work

## Data Availability

The original contributions presented in this study are included in this article and supplementary material. Further inquiries can be directed to the corresponding authors.

## Supplementary Materials

The following supporting information can be downloaded at: https://www.mdpi.com/article/10.3390/toxins16120515/s1, Figure S1: Calibration curve of AFM1 obtained following the anchored primary antibody assay configuration; Table S1: Recovery of known amounts of AFM1 spiked in full-fat cow milk from three different Greek dairy companies.

## References

[1] El-Sayed R.A., Jebur A.B., Kang W., El-Demerdash F.M. (2022). An Overview on the Major Mycotoxins in Food Products: Characteristics, Toxicity, and Analysis. J. Future Food.

[2] Khan R., Anwar F., Ghazali F.M. (2024). A Comprehensive Review of Mycotoxins: Toxicology, Detection, and Effective Mitigation Approaches. Heliyon.

[3] Szelenberger R., Cichoń N., Zajaczkowski W., Bijak M. (2024). Application of Biosensors for the Detection of Mycotoxins for the Improvement of Food Safety. Toxins.

[4] Kafle S., Paudel M., Shrestha C., Kathayat K.B., Sapkota R.C., Tiwari A., Subedi D. (2024). Aflatoxin M1 Contamination in Dairy Milk in Kathmandu, Nepal. Toxins.

[5] Zentai A., Jóźwiak Á., Süth M., Farkas Z. (2023). Carry-Over of Aflatoxin B1 from Feed to Cow Milk—A Review. Toxins.

[6] Alshannaq A., Yu J.H. (2017). Occurrence, Toxicity, and Analysis of Major Mycotoxins in Food. Int. J. Environ. Res. Public Health.

[7] Galvano F., Galofaro V., Galvano G. (1996). Occurrence and Stability of Aflatoxin M1 in Milk and Milk Products: A Worldwide Review. J. Food Prot..

[8] Kumar P., Mahato D.K., Kamle M., Mohanta T.K., Kang S.G. (2017). Aflatoxins: A Global Concern for Food Safety, Human Health and Their Management. Front. Microbiol..

[9] Sipos P., Peles F., Brassó D.L., Béri B., Pusztahelyi T., Pócsi I., Győri Z. (2021). Physical and Chemical Methods for Reduction in Aflatoxin Content of Feed and Food. Toxins.

[10] (2006). European Commission (EC) Commission Regulation (EU) No 1881/2006 of 19 December 2006 Setting Maximum Levels for Certain Contamination in Foodstuffs as Regards Aflatoxin. Off. J. Eur. Union L.

[11] Chen M., Liu X., Yang S., Chen Z., Di B., Liu W., Yan H. (2022). HPLC–MS/MS Method for the Simultaneous Determination of Aflatoxins in Blood: Toxicokinetics of Aflatoxin B1 and Aflatoxin M1 in Rats. J. Anal. Sci. Technol..

[12] Herzallah S.M. (2009). Determination of Aflatoxins in Eggs, Milk, Meat and Meat Products Using HPLC Fluorescent and UV Detectors. Food Chem..

[13] Wood J.E., Gill B.D., Indyk H.E., Rhemrev R., Pazdanska M., Mackay N., Marley E. (2021). Determination of Aflatoxin M1 in Liquid Milk, Cheese, and Selected Milk Proteins by Automated Online Immunoaffinity Cleanup with Liquid Chromatography‒Fluorescence Detection. J. AOAC Int..

[14] Maggira M., Ioannidou M., Sakaridis I., Samouris G. (2021). Determination of Aflatoxin M1 in Raw Milk Using an HPLC-FL Method in Comparison with Commercial ELISA Kits—Application in Raw Milk Samples from Various Regions of Greece. Vet. Sci..

[15] Iqbal S.Z., Jinap S., Pirouz A.A., Ahmad Faizal A.R. (2015). Aflatoxin M1 in Milk and Dairy Products, Occurrence and Recent Challenges: A Review. Trends Food. Sci. Technol..

[16] Tarannum N., Nipa M.N., Das S., Parveen S. (2020). Aflatoxin M1 Detection by ELISA in Raw and Processed Milk in Bangladesh. Toxicol. Rep..

[17] Kos J., Hajnal E.J., Jajić I., Krstović S., Mastilović J., Šarić B., Jovanov P. (2016). Comparison of ELISA, HPLC-FLD and HPLC-MS/MS Methods for Determination of Aflatoxin M1 in Natural Contaminated Milk Samples. Acta Chim. Slov..

[18] Ferrari L., Rizzi N., Grandi E., Clerici E., Tirloni E., Stella S., Bernardi C.E.M., Pinotti L. (2023). Compliance between Food and Feed Safety: Eight-Year Survey (2013–2021) of Aflatoxin M1 in Raw Milk and Aflatoxin B1 in Feed in Northern Italy. Toxins.

[19] Liu B.H., Chu K.C., Yu F.Y. (2016). Novel Monoclonal Antibody-Based Sensitive Enzyme-Linked Immunosorbent Assay and Rapid Immunochromatographic Strip for Detecting Aflatoxin M1 in Milk. Food Control.

[20] Tang Y., Ma P., Khan I.M., Cao W., Yin Zhang Y., Wang Z. (2024). Lateral flow assay for simultaneous detection of multiple mycotoxins using nanozyme to amplify signals. Food Chem..

[21] Thurner F., Alatraktchi F.A.Z. (2023). Recent Advances in Electrochemical Biosensing of Aflatoxin M1 in Milk—A Mini Review. Microchem. J..

[22] Angelopoulou M., Petrou P., Kakabakos S. (2024). Advances in Interferometric Sensors for the Detection of Food Contaminants. TrAC Trend Anal. Chem..

[23] Radoi A., Targa M., Prieto-Simon B., Marty J.L. (2008). Enzyme-Linked Immunosorbent Assay (ELISA) Based on Superparamagnetic Nanoparticles for Aflatoxin M1 Detection. Talanta.

[24] Pang Y.H., Guo L.L., Shen X.F., Yang N.C., Yang C. (2020). Rolling Circle Amplified DNAzyme Followed with Covalent Organic Frameworks: Cascade Signal Amplification of Electrochemical ELISA for Alfatoxin M1 Sensing. Electrochim. Acta.

[25] Cai C., Zhang Q., Nidiaye S., Yan H., Zhang W., Tang X., Li P. (2021). Development of a Specific Anti-Idiotypic Nanobody for Monitoring Aflatoxin M1 in Milk and Dairy Products. Microchem. J..

[26] Cai C., Liu Y., Tang X., Zhang W., Zhang Q., Li P. (2023). Development of a Toxin-Free Competitive Immunoassay for Aflatoxin M1 Based on a Nanobody as Surrogate Calibrator. LWT.

[27] Capodicasa C., Bastiani E., Serra T., Anfossi L., Catellani M. (2022). Design of a Diagnostic Immunoassay for Aflatoxin M1 Based on a Plant-Produced Antibody. Toxins.

[28] Tsounidi D., Koukouvinos G., Petrou P., Misiakos K., Zisis G., Goustouridis D., Raptis I., Kakabakos S.E. (2019). Rapid and Sensitive Label-Free Determination of Aflatoxin M1 Levels in Milk through a White Light Reflectance Spectroscopy Immunosensor. Sens. Actuator B Chem..

