# Supplementary material for: Sensitive Aflatoxin M1 Detection in Milk by ELISA: Investigation of Different Assay Configurations
Source: Toxins (Basel). 2024 Nov 29;16(12):515. doi: 10.3390/toxins16120515 (PMC11728486; doi:10.3390/toxins16120515)
Supplement: Supplementary file 1 [file toxins-16-00515-s001.zip › toxins-3329101-supplementary.pdf]

toxins

MDPI

Supplementary material

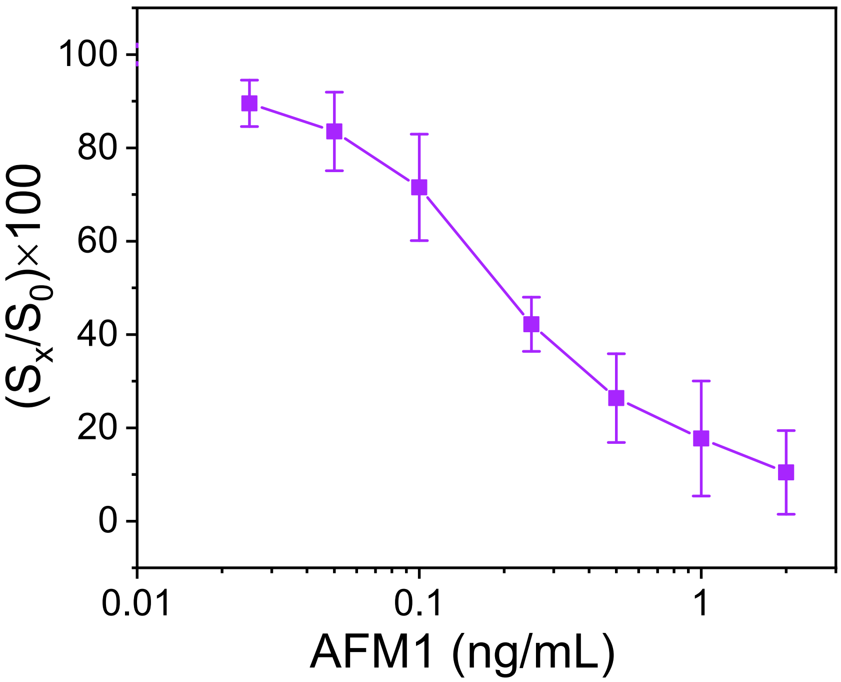

Figure S1. Calibration curve of AFM1 obtained following the anchored primary antibody assay configuration.

Table S1. Recovery of known amounts of AFM1 spiked in full fat cow milk from three different Greek dairy companies.

Sample	Amount added (pg/mL)	Amount determined (pg/mL)	%Recovery
**1**	40	42	105
	120	110	91.7
	400	360	90.0
	1200	1300	108
**2**	40	36	90.0
	120	130	108
	400	370	92.5
	1200	1100	91.7
**3**	40	43	108
	120	105	87.5
	400	420	105
	1200	1250	104
